# Oligomeric Amyloid‐β, Tau Pathology, and Glucose Dysregulation in Alzheimer’s Disease: Uncovering Early Metabolic Vulnerability Associated with Soluble Aβ Species

**DOI:** 10.1002/alz70861_107940

**Published:** 2025-12-23

**Authors:** Dong Woo Kang

**Affiliations:** ^1^ College of Medicine, Seoul St. Mary's Hospital, The Catholic University of Korea, Seoul, Seoul Korea, Republic of (South)

## Abstract

**Background:**

Alzheimer’s disease (AD) is increasingly conceptualized as “Type 3 Diabetes” (T3D) due to its association with insulin resistance and glucose metabolic dysfunction. Among the core pathological features of AD, oligomeric amyloid‐β (Aβ) has emerged as a potent mediator of neuronal and metabolic toxicity, yet its differential role compared to fibrillar Aβ plaques in systemic glucose dysregulation remains unclear.

**Method:**

This cross‐sectional study evaluated 113 participants across the cognitive spectrum, including cognitively normal older adults, patients with mild cognitive impairment, and Aβ‐PET‐positive dementia. Plasma oligomeric Aβ levels were measured using the Multimer Detection System (MDS‐OAβ), and tau pathology was assessed through [18F]‐flortaucipir PET using Braak staging. Glucose metabolic indices included hemoglobin A1c (HbA1c) and fasting plasma glucose levels. Generalized linear models (GLMs) were used to examine main and interaction effects between MDS‐OAβ burden and tau pathology on glucose regulation, adjusting for age, sex, education, APOE ε4 status, antidiabetic medication use, and global CDR score.

**Result:**

A significant interaction between MDS‐OAβ risk and tau Braak stage was observed for HbA1c levels (χ² = 22.177, *p* = 0.001), but not for fasting glucose. Post‐hoc analyses revealed that individuals at Braak stage 0 with high MDS‐OAβ burden exhibited significantly elevated HbA1c levels compared to those with lower Aβ oligomer levels (Bonferroni‐adjusted *p* < 0.001). In contrast, no significant metabolic associations were identified with Aβ‐PET SUVR, and no significant interactions were observed between continuous AD biomarkers and tau‐PET SUVR. These results suggest a tauopathy‐dependent effect of soluble Aβ species on long‐term glucose dysregulation during early disease stages.

**Conclusion:**

Oligomeric Aβ burden, but not fibrillar amyloid deposition, was associated with elevated HbA1c levels in individuals without overt tau accumulation, indicating an early metabolic vulnerability within the T3D framework. These findings support the clinical relevance of differentiating soluble from fibrillar Aβ species when evaluating AD‐related metabolic dysfunction and highlight the importance of staging tau pathology in biomarker‐based models of disease progression.

